# Stereotactic Radiosurgery and Highly Active Antiretroviral Therapy for HIV-Related Primary Central Nervous System Lymphomas: A Prospective Study Analyzing the Efficacy and Safety

**DOI:** 10.1227/neuprac.0000000000000072

**Published:** 2023-12-07

**Authors:** Andres M. Alvarez Pinzon, Jose Ramon Alonso, Aizik Wolf, Felipe Ramirez-Velandia, Jose E. Valerio

**Affiliations:** * The Institute of Neuroscience of Castilla y León (INCYL), Cancer Neuroscience, Universidad de Salamanca (USAL), Salamanca, Spain;; ‡ Department of Neurological Surgery, Latino America Valerio Foundation, Weston, Florida, USA;; § Institute for Human Health and Disease Intervention (I-HEALTH), Florida Atlantic University, Jupiter, Florida, USA;; ‖ LEAD Program, Graduate School of Business, Stanford University, Palo Alto, California, USA;; ¶ The Institute of Neuroscience of Castilla y León (INCYL), University of Salamanca (USAL), Salamanca, Spain;; ** Department of Neurosurgery, Miami Neuroscience Center at Larkin, Neurosurgery Oncology Center of Excellence, Miami, Florida, USA;; ‡‡ Department of Neurological Surgery, Palmetto General Hospital, Miami, Florida, USA

**Keywords:** AIDS, Gamma knife radiosurgery, HIV, Primary central nervous system lymphoma, Stereotactic radiosurgery

## Abstract

**BACKGROUND AND OBJECTIVES:**

Stereotactic radiosurgery (SRS) has been well established and considered a safe alternative for primary central nervous system lymphomas (PCNSLs). However, in HIV-related PCNSL, the use of SRS remains controversial. The purpose of this study was to evaluate the efficacy and safety of SRS in HIV-related PCNSL.

**METHODS:**

Forty-two adult patients with confirmed PCNSL and no previous radiotherapy or chemotherapy were selected, with 16 receiving Gamma Knife Radiosurgery (GKRS) alone and 26 receiving Highly Active Antiretroviral Therapy (HAART) before GKRS. Follow-up evaluations were conducted at 3, 6, 12, and 24 months post-treatment using clinical and imaging techniques. Statistical analyses were performed using SPSS v22, assessing for new lesions, changes in lesion size, progression-free survival, and overall survival.

**RESULTS:**

HAART/GKRS showed a significantly higher rate of complete response compared with the GKRS group, with 53.8% vs 18.8% (*P* = .048). The mean progression-free survival for the HAART/GKRS group and the GKRS group was 39.7 months and 31.2 months, respectively (*P* = .0051). Patients with a delayed HAART initiation (>6 months) exhibited an increased burden of T2 white matter lesions and a higher number of large lesions (odds ratio = 1.9, 95% CI = 1.44-2.42, *P* = .001). However, no significant difference was observed between the two groups regarding radionecrosis.

**CONCLUSION:**

The study highlights the potential benefits of adding HAART to GKRS for patients with PCNSL, leading to improved survival outcomes. An early initiation of HAART was associated with less tumor progression, underscoring the importance of timely administration of HAART in patients with AIDS-related PCNSL.

**ABBREVIATIONS:**
CR: complete response
EBV: Epstein-Barr virus
ECOG: Eastern Cooperative Oncology Group
HAART: Highly Active Antiretroviral Therapy
LDH: lactate dehydrogenase
LCR: light chain restriction
NA: nonapplicable
ORR: overall response rate
OS: overall survival
PD: progressive disease
PCNSL: primary central nervous system lymphoma
PR: partial response
RS: radiosurgery
SD: stable disease
SRS: stereotactic radiosurgery
WBRT: whole-brain radiotherapy.

Primary central nervous system lymphomas (PCNSLs) are aggressive and uncommon brain tumors that account for 4% of primary central nervous system neoplasms.^1,2^ PCNSL routinely presents as a supratentorial single or multifocal contrast-enhancing lesions, but the definitive diagnosis of PCNSL relies on histopathological confirmation.^3^

Patients with HIV are much more prone to PCNSL because it is suspected that the coinfection with Epstein-Barr virus (EBV) can trigger oncogenic proliferation of lymphocytes, causing large or immunoblastic non-Hodgkin's B-cell lymphomas.^4^ Despite this, since the introduction of Highly Active Antiretroviral Therapy (HAART) in 1996, the rate of AIDS has decreased dramatically, including AIDS-defining cancers such as PCNSL.^5^

In comparison with other brain tumors, resection is not indicated for the treatment of newly diagnosed HIV-PCNSL. First-choice treatment is the combination of HAART with high-dose methotrexate (MTX).^6^ Other alternatives that have recently shown worthy results are HAART combined with whole-brain radiotherapy (WBRT) and high-dose MTX with gamma knife radiosurgery (GKRS) in the immunocompetent population.^7^ The median survival of untreated PCNSL is poor, varying from 2 to 4 months, but chemotherapy treatment prolongs the median survival to 1.5 years.^8^

Stereotactic radiosurgery (SRS) is a noninvasive treatment that uses beams of gamma radiation to target a specific lesion. The use of SRS for brain tumors has been established as a treatment option for metastatic disease, meningiomas, pituitary adenomas, and vestibular schwannomas,^9^ but its use for other types of tumors (as in PCNSL) has been reserved as a last resource or stopgap. Some studies have considered SRS a safe alternative for the treatment of PCNSL, showing improvement in median survival, symptoms, and quality of life.^7,10-12^ However, the role of SRS in the initial management of HIV-PCNSL remains controversial.

Therefore, the primary objective of the study was to assess tumor response and survival rates of SRS in HIV-related PCNSL. In addition, the study aims to evaluate the effect of HAART on tumor response and clinical outcomes in SRS management.

## METHODS

### Study Design

This prospective observational study enrolled 42 adult patients with confirmed PCNSL and no previous radiotherapy or chemotherapy. Patients were selected based on physician discretion and provided informed consent following Institutional Review Board approval and in accordance with the Strengthening the Reporting of Observational Studies in Epidemiology guidelines.

### Setting

The study was conducted as part of The Brain Tumor Registry Study between June 2010 and August 2017, with retrospective assessments from January 2012 to June 2016 and prospective assessments from June 2016 to June 2019; the final follow-up occurred in July 2023.

### Participants

Participants were eligible for this study if they met the following criteria: (1) had a confirmed diagnosis of PCNSL by stereotactic brain tumor biopsy confirmed by an experienced neuropathologist, (2) had not received radiotherapy, (3) had been diagnosed with AIDS, (4) were at least 18 years old, and (5) had no history of cancer treatment. AIDS diagnosis was confirmed according to the Centers for Disease Control and Prevention's stringent criteria,^13^ which requires a positive HIV blood test and a CD4 count of less than 200 cells per cubic millimeter of blood (200/mm^3^) or a major opportunistic condition, such as certain types of cancer, infections, and syndromes that are frequently associated with AIDS. The Department of Infectious Diseases and Pathology reviewed and validated all HIV-related laboratory data and confirmed cases.

### Variables

The study considered several variables, including age, sex, initial presenting symptoms, functionality, tumor response, survival rates, treatment regimens (GKRS alone, GKRS + HAART), and the timing of HAART administration (early or late). HAART was considered a 3-antiretroviral regimen that includes at least two different groups of drugs, including a nucleoside reverse transcriptase inhibitor and a non-nucleoside reverse transcriptase inhibitor. Early treatment was defined as HAART initiation within 6 months of PCNSL diagnosis, whereas late treatment was considered if the treatment was started beyond this timeframe. For those patients who underwent GKRS and HAART, the administration of HAART was initiated at least 28 days before GKRS.

The tumor response was evaluated based on the modified Response Criteria of the International PCNSL Collaboration Group.^14^ Complete Response (CR) was defined as the complete disappearance of all evidenced lesions; partial response was defined as a reduction in tumor size of 50% or more. Progressive disease (PD) was defined as an increase in tumor size by 25% or more or the development of a new lesions. Stable disease (SD) was defined as any patient outcome that did not meet any of the abovementioned criteria. In addition, progression-free survival was defined as the time from diagnosis to recurrence of the disease, progression of the disease, or death.

### Data Sources/Measurement

Comprehensive clinical data were collected from patients diagnosed with PCNSL through in-person interviews and medical examinations at specific timepoints: before treatment and 3, 6, 12, and 24 months post-treatment. The presenting symptoms, including seizures, focal neurological deficits, headaches, and baseline functionality assessed using the Karnofsky Performance Status and Eastern Cooperative Oncology Group Performance Scale, were documented. All participants underwent MRI with volume T1 acquisition using the Magnetization Prepared Rapid Acquisition Gradient Echo technique with 1-mm slice reconstruction before treatment and 3 months after treatment.

GKRS was performed using the Leksell Gamma Knife® PerfexionTM. After the neurosurgeon placed the frame, the patients underwent a high-resolution, thin-slice MRI with contrast (1.5 T) to enhance target accuracy in the GammaPlan treatment planning software. Treatment planning integrated 1-mm axial slice thickness MRI with CT scans. In cases where T1 imaging was insufficient, fluid-attenuated inversion-recovery sequences were used for complete tumor delineation. Prescribed doses ranged from 10 to 20 Gy, following the protocol established by the Miami Neuroscience Center. The doses were adjusted following an inverse square function, with decreasing doses administered as the target volume increased. Dose planning involved the use of multiple isocenters to optimize the dose-gradient index, with the prescription set at the 50% isodose line, and no augmentation was made to the gross tumor volume. Treatment plans received approval from both a radiation oncologist and a neurosurgeon before implementation. Dosage and treatment volume were meticulously documented for each treated lesion.

### Bias

A single-blind data collection using REDCap was implemented with the assistance of research fellows to decrease the performance bias in the study.

### Study Size

**FIGURE 1.**
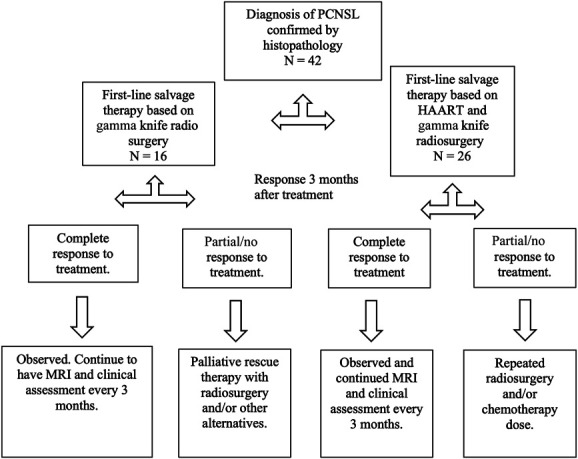
Clinical protocol diagram. The flowchart depicts the patients included in the analysis and their classification based on their treatment response after 3 months. The objective of this study was to compare the efficacy of GKRS alone vs GKRS in combination with HAART for the treatment of PCNSL associated with AIDS. GKRS, Gamma knife radiosurgery; HAART, Highly Active Antiretroviral Therapy; PCNSL, primary central nervous system lymphoma.

Of the 42 patients included in the study based on the selection criteria, the sample was divided into two groups: those who received GKRS + HAART and those who received GKRS alone (Figure 1).

### Quantitative Variables

In addition to the previously mentioned variables, the study included additional quantitative variables like serum lactate dehydrogenase, Light Chain Restriction protein, CD4 counts, and viral load. To assess the normality of the distribution of these quantitative variables, statistical normality tests, such as the Skew test and Shapiro–Wilk test, were used.

### Statistical Methods

Statistical analysis was performed using SPSS version 22 (SPSS, Inc.). Specifically, the analyses aimed to evaluate new lesions at the tumor level, changes in lesion size or disappearance, progression-free survival, and overall survival (OS). Secondary end points such as toxicity at the clinical level and comorbidities were also evaluated through data analysis. Quantitative and ordinal variables were compared using Mann–Whitney tests. Furthermore, the Kaplan–Meier method was used for the univariate analysis of survival. Differences in survival rates were assessed using the log-rank test. The CIs for survival times were constructed from the logarithmic transform of the Kaplan–Meier survival estimator (product limit). All reported *P*-values were bilateral, and statistical significance was defined as *P* < .05.

## RESULTS

### Participants and Descriptive Data

In the study, 42 patients with AIDS-related PCNSL were included, with 16 receiving GKRS and 26 receiving GKRS plus HAART (Figure 1). There were more males in both groups, and the average age of patients in the GKRS and GKRS plus HAART groups was 51 and 54 years, respectively, with no statistically significant difference (*P* = .349). Baseline functionality, tumor burden, CD4 count, viral load, lactate dehydrogenase levels, and Light Chain Restriction protein levels were similar between the two groups, indicating a well-balanced comparison at baseline (Table 1).

**TABLE 1.** Basic Characteristics of the Patients Included for the Analysis

Characteristics	GKRS (n = 16)	GKRS + HAART (n = 26)	*P* value
Average age, years (range)	51 (30-67)	54 (16-69)	.349
Male	11	17	.51
Female	5	9		
Average ECOG performance status scale (range)	3 (2-4)	3 (2-4)		.901
High LDH	9	13		.879
High LCR protein	12	19		.59
Multiple lesions	14	23		.37
Average lymphocyte CD4 cell in mm^3^	131 mm^3^	123 mm^3^		.73
Viral load >55.000	16	24		.96

ECOG, Eastern Cooperative Oncology Group; GKRS, Gamma knife radiosurgery; HAART, Highly Active Antiretroviral Therapy; LDH, lactate dehydrogenase; LCR, light chain restriction.

### Outcome Data

The main objective of this study was to evaluate the tumor response according to the treatment regimen. We found that in patients with AIDS-related PCNSL, the addition of HAART to GKRS resulted in a significantly higher rate of CR compared with GKRS alone (53.8% vs 18.8%, *P* = .048). Although the PD rate was higher in the GKRS group, the difference was not statistically significant (*P* = .126). The overall response rates were similar for both groups (84.6% for GKRS plus HAART and 87.5% for GKRS), with no significant difference (*P* = .979) (refer to Table 2).

**TABLE 2.** Tumor Response in GKRS vs GKRS + HAART Groups

RECIL	Number of patients, N (%)		*P* value
	GKRS (n = 16)	GKRS + HAART (n = 26)	
CR	3 (18.8)	14 (53.8)	.048
PR	9 (56.25)	11 (69.2)	.483
ORR	14 (87.5)	22 (84.6)	.979
SD	2 (12.5)	2 (7.7)	NA
PD	4 (25)	1 (3.8)	.126

CR, complete response; GKRS, Gamma knife radiosurgery; HAART, Highly Active Antiretroviral Therapy; NA, nonapplicable; ORR, overall response rate; PD, progressive disease or general response rate; PR, partial response; RECIL, Response Evaluation Criteria In Lymphoma; SD, stable disease.

In our clinical analysis, we observed that the median survival time for both patient groups was 34.1 months. However, it is noteworthy that the group receiving HAART in addition to GKRS demonstrated a significantly higher survival rate, with a median survival of 39.7 months, compared with the GKRS-alone group, which had a median survival of 31.2 months (*P* = .0051). Table 3 compares the median survival of different treatment cohorts for PCNSL. The HAART + GKRS group from this study had the highest median survival. Other studies included MTX + radiosurgery (RS), MTX or WBRT + RS, and systemic chemotherapy, with lower median survival rates.^10-12^

**TABLE 3.** Comparison of Survival According to the Therapy in Other Similar Cohorts

Treatment and study	Median survival (mo)
HAART + GKRS (our analysis)	39.7
MTX + RS (Alvarez et al)^10^	47.6
MTX or WBRT + RS (Kenai et al)^11^	32.1
Chemotherapy (Adam et al)^12^	18

GKRS, Gamma knife radiosurgery; HAART, Highly Active Antiretroviral Therapy; MTX, methotrexate; RS, radiosurgery; WBRT, whole-brain radiotherapy.

### Main Results

When evaluating the timing of HAART administration, we found that initiating HAART early resulted in a significant 40% reduction in HIV load (25% to 52%). Furthermore, a positive correlation was observed between tumor volume and delayed initiation of HAART (odds ratio = 1.9, 95% CI = 1.44-2.42, *P* < .001). Similarly, there was an increased burden of T2 white matter lesions in patients who initiated HAART late. This suggests that a delayed start of HAART was associated with larger tumor volumes and a higher number of lesions in patients with PCNSL. Moreover, after a 3-month follow-up period, we observed that a subset of patients (23 in total) exhibited a CD4 count below 200 IU, with a notable difference between the two groups. Specifically, 17 patients from the GKRS group had a CD4 count below this threshold, whereas only four patients from the GKRS + HAART group fell into the same category (*P* = .00291).

Regarding brain MRI analysis of patients diagnosed with PCNSL, several critical findings emerged. Most of these patients exhibited a rather distinctive MRI profile characterized by the presence of multiple lesions, with the majority having more than four distinct lesions. These lesions were prominently marked by well-defined enhancing T1 lesions. Notably, our study revealed considerable variability in the number of lesions, with an average of 13 lesions per patient, and at least three of these lesions were larger than 2 cm.

In our recurrence rate analysis, we found that 13 patients from the GKRS group and nine patients from the GKRS + HAART group experienced recurrences. Intriguingly, our analysis identified a correlation between CD4 count levels and recurrence risk. Specifically, patients who exhibited a CD4 count lower than 200 IU/mm^3^ after a 3-month follow-up period were more susceptible to tumor recurrence. These observations shed light on the potential influence of immunological factors, particularly CD4 counts, in shaping the course of PCNSL progression and recurrence.

**FIGURE 2.**
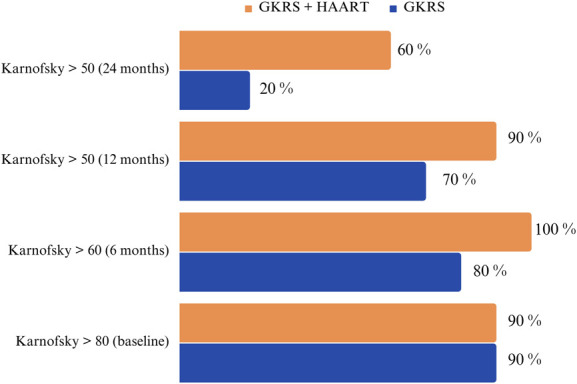
Karnofsky scale evaluation during follow-up in the GKRS + HAART group compared with the GKRS group. GKRS, Gamma knife radiosurgery; HAART, Highly Active Antiretroviral Therapy.

Our assessment of patients' functionality, as measured by the Karnofsky scale (Figure 2), revealed a promising trend in favor of the GKRS + HAART group compared with the GKRS-alone group. This trend suggests that the combined therapy not only positively influences survival rates but also contributes to improved overall well-being and functional capacity in patients with PCNSL.

### Other Analyses

**FIGURE 3.**
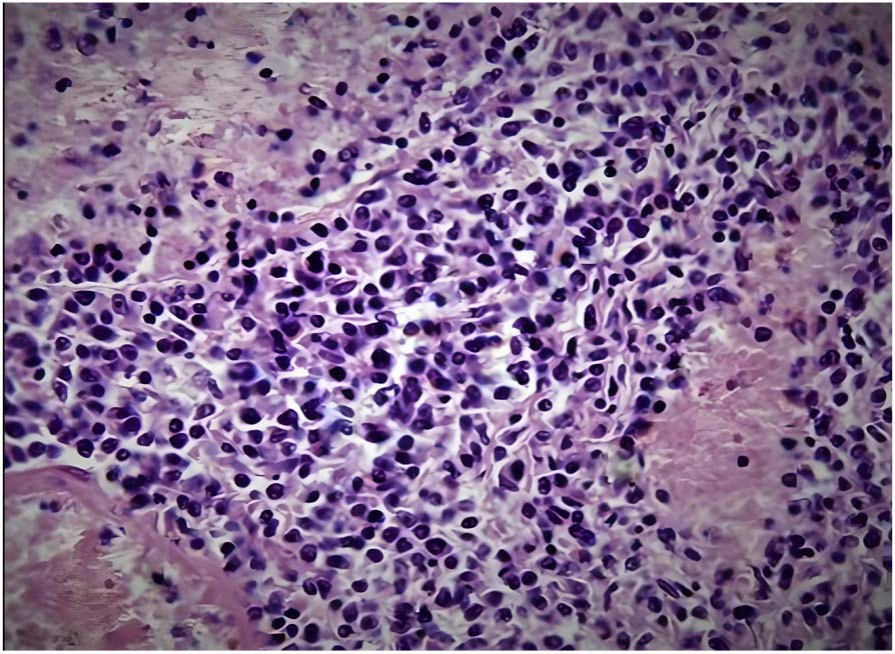
Primary central nervous system lymphoma, Hematoxylin and Eosin × 400 staining. The induction of Epstein-Barr virus and HIV from latency can render tumor cells susceptible to antiviral treatment with agents used in highly active antiretroviral therapy and, consequently, more susceptible to radiosurgery.

Histopathology analysis of tumor samples from 36 patients (86%) revealed a latent EBV infection in tumor cells, and there was no significant difference between the two treatment groups. In addition, all patients exhibited the characteristic angio/centricity and angioinvasion features associated with PCNSL (Figure 3).

**FIGURE 4.**
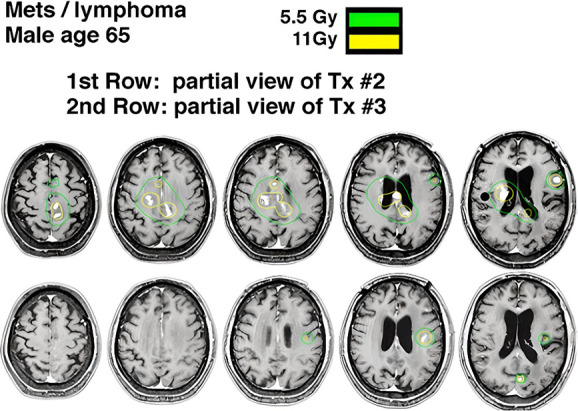
Illustrative radiosurgical plan is detailed for TX #2 and TX #3, demonstrating local tumor control in patients diagnosed with PCNSL. This case involves a 65-year-old male presenting with progressive PCNSL and AIDS. MRI with a 2 mm expansion from gross tumor volume to planning target volume, administering a prescription dose of 11 Gy. Subsequent to a nine-month interval, follow-up MRI images reveal the patient's resolution of enhancing disease. In TX #3, additional lesions were addressed and treated with a resultant complete resolution.

In regard to toxicity, no significant difference was found between the two treatment groups concerning radionecrosis. Patients with radionecrosis had larger tumor volumes (average 3.3 cm^3^) compared with those without radionecrosis (average 1.9 cm^3^). The incidence rate of radionecrosis correlated with tumor diameter. Tumors ≤0.5 cm had a low incidence rate of 1.1% (95% CI 0.6%-7%), whereas tumors >1.5 cm had a higher incidence rate of 47.0% (95% CI 25.9%-52.8%). A detailed plan of GKRS treatment before and after is shown in Figure 4. No major side effects, such as motor or language deficits, were observed in any patients.

## DISCUSSION

Over the past decade, GKRS has found increasing applications in neurosurgery, with mounting evidence of clinical and volumetric responses in various tumors and vascular malformations. Specifically, for AIDS-associated PCNSL, GKRS has emerged as an effective alternative to WBRT and chemotherapy because of its minimal toxicity and favorable overall response rates, as demonstrated in previous studies.^15-19^

In our analysis, we observed that combining HAART with GKRS resulted in a statistically significant reduction in tumor burden, a higher rate of CR to treatment, and an improvement in quality of life as evaluated by the Karnofsky scale. In addition, we report a median survival rate of 39.7 months for the HAART + GKRS group, which is comparable with the outcomes of other SRS-based treatments for PCNSL (refer to Table 3 and summary of key findings provided in Table 4).

**TABLE 4.** Clinical Research Outcomes in AIDS-Related PCNSL

Results summary	HAART + GKRS	GKRS alone
CR rate	53.8%	18.8%
ORR	84.6%	87.5%
PD rate	Higher	—
HIV load reduction with early HAART initiation (range)	40% (25%-52%)	—
Tumor volume correlation with delayed HAART initiation	Positive	—
Median survival time (mo)	39.7	31.2
CD4 count below 200 IU after 3 mo	Fewer	More
Brain MRI findings	Multiple lesions, well-defined enhancing T1 lesions, average 13 lesions/patient	—
Tumor recurrence correlation with CD4 count <200 IU/mm^3^	Yes	—

The following points summarize the clinical outcomes of our study: CR rate significantly improved with HAART + GKRS No significant difference in ORR between the two groups PD rate was higher in the GKRS group but not statistically significant Early HAART reduced HIV load, and delayed initiation correlated with larger tumor volumes and more lesions GKRS + HAART group exhibited higher median survival and better Karnofsky scores Fewer patients had CD4 counts below 200 IU after 3 months with HAART + GKRS Brain MRI revealed multiple lesions, mostly >4, with tumor recurrence correlated with low CD4 counts Radionecrosis incidence was not significantly different One case of asymptomatic AVM in the GKRS group HAART + GKRS group showed the highest median survival compared with other PCNSL treatment cohorts

AVM, arteriovenous malformation; CR, complete response; GKRS, Gamma knife radiosurgery; HAART, Highly Active Antiretroviral Therapy; ORR, overall response rate; PCNSL, primary central nervous system lymphomas; PD, progressive disease.

It is well known that optimal management of AIDS-related PCNSL requires controlling the HIV viral load and CD4 count with HAART. Therefore, early initiation of HAART plays a pivotal role in achieving disease remission, curtailing viral load, and enhancing OS in patients with AIDS-related PCNSL.^20^ Our study revealed that patients who delayed HAART initiation by more than 6 months exhibited a notable increase in T2 white matter lesions and displayed larger lesions on imaging. This observation is consistent with the hypothesis that HAART-induced immune reconstitution may effectively ameliorate polyclonal EBV and HIV-induced lymphoproliferation that has been implicated in the tumor pathogenesis.^19,21^ EBV has been extensively studied because of its pro-oncogenic potential, and latent infection was identified in 86% of our patients. Efforts are increasing to use ganciclovir as a treatment strategy to improve survival in PCNSL, and the use of ganciclovir in combination with HAART has shown promising results in various cohorts.^22,23^ However, the combination of ganciclovir, SRS, and HAART remains an unexplored avenue that warrants investigation.

In a separate publication by Wu et al,^24^ a retrospective review was conducted on 20 patients with PCNSL who underwent single-fraction or fractionated SRS for a total of 32 lesions over a span from September 1992 to July 2019. The median age at SRS was 67 years (IQR = 56-74 years), and the median time to distant failure post-SRS was 10 months (IQR = 1-16 months). Salvage SRS or WBRT was administered to address progression in select cases. The study found that Karnofsky Performance Status at the time of SRS significantly correlated with the time to progression (*P* = .002). Furthermore, the utilization of lenalidomide or pomalidomide post-SRS was associated with improved OS compared with those who did not receive these agents (3 vs 14 months, *P* = .035). In addition, consolidative etoposide and cytarabine after initial methotrexate-based chemotherapy demonstrated a positive impact on survival after SRS (8 vs 47 months, *P* = .028). These findings suggest that SRS effectively ensures local tumor control in PCNSL when given as a salvage therapy post-WBRT or as an option to defer WBRT in specific cases. However, most of the patients in this cohort experienced distant progression. Nevertheless, it is important to note that systemic therapy seems to exert a substantial influence on outcomes in this patient cohort.

Within our patient cohort, the combination therapy of HAART and GKRS for PCNSL exhibited a noteworthy enhancement in patients' performance status, as evident from improvements in their Karnofsky scores (Figure 3). This observation is consistent with previous investigations that have underscored the positive impact of GKRS on the overall well-being of patients with PCNSL.^25-27^ However, it remains essential to acknowledge persistent concerns regarding the limited treatment volume offered by SRS and its potential implications for long-term disease control.^18^ These considerations emphasize the imperative for continuous research aimed at refining treatment strategies for PCNSL.

In our study, the HAART + GKRS group demonstrated a significantly higher rate of CR compared with the other group, whereas no notable differences were observed in partial response, PD, or SD. These findings are in concordance with a recent meta-analysis, which reported CR in all patients with PCNSL treated with SRS, with minimal instances of PD or distant recurrence.^17^ In addition, corroborating studies have also documented improvements in cognitive function and performance status among patients with PCNSL after SRS treatment.^28-31^ Taken together, these findings underscore the effectiveness and safety of SRS as a treatment modality, not only improving prognosis and reducing tumor volume but also enhancing short-term clinical outcomes in PCNSL.

An intriguing avenue of investigation involves determining whether a discernible variance in response to GKRS exists contingent upon the specific anatomic location of each lesion. Given the multifocal nature of PCNSL, it presents a significant challenge when assessing both tumor location and response rates. Notably, previous reports have suggested a worse prognosis for patients with PCNSL with infratentorial lesions or volumes exceeding 11.4 cm.^31^ Consequently, further investigation in future studies is particularly compelling because of the limited information available in this area.

### Limitations

Our study has several limitations that should be acknowledged. First, the heterogeneity of the study population and the absence of randomization introduce the possibility of selection bias, which may affect the generalizability of our findings. Second, we were unable to evaluate the effectiveness of salvage treatments used during the course of treatment, including immunotherapy, newer chemotherapy regimens, or targeted therapies, which could have influenced patient outcomes. Despite the limitations, our study's results align with previous research and contribute valuable insights into the potential advantages of combining HAART with GKRS in the management of PCNSL.

## CONCLUSION

In conclusion, our study sheds light on the promising role of GKRS as a noninvasive treatment modality for the management of HIV-associated PCNSL, particularly when combined with HAART. Our findings indicate that this therapeutic approach offers potential advantages in tumor control, prolonged survival, and an overall improvement in the quality of life of affected patients. However, it is imperative to acknowledge the necessity for further research to refine our understanding of the specific patient subgroups that would derive the greatest benefits from GKRS. In addition, comparative studies evaluating the effectiveness of GKRS/HAART against other treatment modalities, with a focus on prognosis and long-term recurrence rates, are warranted. Furthermore, exploring the potential synergistic effects of combining GKRS/HAART with other EBV treatment strategies represents a promising avenue for future investigation. Our study contributes valuable insights to the evolving landscape of HIV-associated PCNSL management, highlighting the potential of GKRS/HAART combination therapy while emphasizing the need for ongoing research and multidisciplinary approaches to optimize patient outcomes in this challenging clinical scenario.
